# Large survey dataset of rice production practices applied by farmers on their largest farm plot during 2018 in India

**DOI:** 10.1016/j.dib.2022.108625

**Published:** 2022-09-20

**Authors:** Anurag Ajay, Peter Craufurd, Virender Kumar, Arindam Samaddar, RK Malik, Sachin Sharma, Harshit Ranjan, AK Singh, Gokul Paudel, Ajay Kumar Pundir, Shishpal Poonia, Anurag Kumar, Pankaj Kumar, Deepak Kumar Singh, Madhulika Singh, Wasim Iftikar, Moben Ignatius, Narayan Banik, Bidhan Mohapatra, Pardeep Sagwal, Ashok Kumar Yadav, Sugandha Munshi, Peramaiyan Panneerselvam, Andrew McDonald

**Affiliations:** a International Maize and Wheat Improvement Center (CIMMYT), Carretera México-Veracruz, Km. 45, El Batán, Texcoco 56237, Mexico; b International Rice Research Institute (IRRI), Pili Drive, Los Baños, Laguna 4031, Philippines; c Indian Council of Agricultural Research (ICAR), Division of Agricultural Extension, Krishi Anusandhan Bhawan, New Delhi 110 012 India; d College of Agriculture and Life Sciences, Cornell University, 617 Bradfield Hall, Ithaca, NY 14853 USA

**Keywords:** Agronomy, Farmer survey, India, Production practices, Rice

## Abstract

This dataset provides detailed information on rice production practices being applied by farmers during 2018 rainy season in India. Data was collected through computer-assisted personal interview of farmers using the digital platform Open Data Kit (ODK). The dataset, *n* = 8355, covers eight Indian states, viz., Andhra Pradesh, Bihar, Chhattisgarh, Haryana, Odisha, Punjab, Uttar Pradesh and West Bengal. Sampling frames were constructed separately for each district within states and farmers were selected randomly. The survey was deployed in 49 districts with a maximum of 210 interviews per district. The digital survey form was available on mobile phones of trained enumerators and was designed to minimize data entry errors.

Each survey captured approximately 225 variables around rice production practices of farmers’ largest plot starting with land preparation, establishment method, crop variety and planting time through to crop yield. Detailed modules captured fertilizer application, irrigation, weed management, biotic and abiotic stresses. Additional information was gathered on household demographics and marketing. Geo-points were recorded for each surveyed plot with an accuracy of <10 m. This dataset is generated to bridge a data-gap in the national system and generates information about the adoption of technologies, as well as enabling prediction and other analytics. It can potentially be the basis for evidence-based agriculture programming by policy makers.

**Specifications Table**

**Table:** 

Subject	Agricultural Sciences
Specific subject area	Agronomic practices of rice farmers at landscape level – from land preparation through all crop management practices and yields
Type of data	Map Table Graph
How the data were acquired	Data was collected by trained enumerators through personal interviews of all the randomly selected rice farmers. Enumerators used digital survey instrument available on their Android-based phone through ODK Collect app. Completed surveys were sent by enumerators to a cloud-based server of ODK called Aggregate. Auto-compiled raw dataset was downloaded from server.
Data format	Curated Anonymized
Description of data collection	District was the survey unit so sampling frame was constructed for each district. From the list of villages fetched from 2011 census data of India, 30 villages were selected randomly using probability-proportionate-to-size method. Extremely small and large villages including all urban habitats were excluded. From each selected village, seven households were selected randomly using electoral roll of the village. Farmers were asked to answer questions regarding their largest rice plot only. There were roughly 225 questions covering land preparation to rice harvesting. All farming practices applied by farmers, viz., rice variety used, time of transplanting, fertilizers, irrigation, herbicides, pesticides, etc. were recorded. All surveyed plots were geo-tagged. During curation, the name of farmers and their contact numbers were deleted, and geo-points of surveyed plots were truncated to two (dd.dd) decimal points from originally six (dd.dddddd).
Data source location	Data were collected largely from eastern part of India including some eastern coastal states. Two states from north-western part were also surveyed. States: Andhra Pradesh, Bihar, Chhattisgarh, Haryana, Odisha, Punjab, Uttar Pradesh, and West Bengal Country: India Latitude and longitude: Truncated geo-location of every rice plot (8,355 plots) surveyed is available in the data file – columns ‘HM’ and ‘HN’.
Data accessibility	The dataset and accompanying files (e.g., R code to read data summary, ODK Build file, maps, variable details, 3Decimal point geo-coordinates, etc.) are open access through CIMMYT's Dataverse webpage. Repository name: CIMMYT Research Data & Software Repository Network (https://data.cimmyt.org/) Data identification number: https://hdl.handle.net/11529/10548656 Direct URL to data: https://data.cimmyt.org/dataset.xhtml?persistentId=hdl:11529/10548656

**Value of the Data**

• This dataset is unique in the data ecosystem of India as it records in detail farmers’ current rice production practices. It can be used as a monitoring tool/feedback mechanism by national agricultural system for site specific technology targeting.
• The dataset is quite large and covers many different geographies/agroecological zones. It generates adequate information to learn how rice cultivation practices vary from place to place within India.
• Farmers for the interviews were selected using purely random method so the information including yields can be very well generalized for a larger geo-political domain. If replicated, concerned agency can generate panel data to assess change in practices and productivity gains over time.
• The data generates information on location-specific usage rate of farm inputs and adoption status of agricultural technologies. It is thus valuable for private sector firms dealing in seeds, fertilizers, herbicides machineries in terms of market development/expansion.
• Crop modelers can layer this data with other datasets (weather, soil, topography, etc.) as every datapoint in this dataset is geo-referenced. It can then be reused in developing algorithms for yield predictions.

## 1 Data Description

This survey dataset [3] from India has large spatial distribution and it provides complete details about ricer production practices. The dataset is diverse since surveyed crop was spread over different cropping as well as production systems. The survey form was kept consistent across states and sites to ensure uniformity of data [1]. A similar dataset was generated earlier for the wheat crop in Bihar and Eastern Uttar Pradesh [2].

**Map 1:**
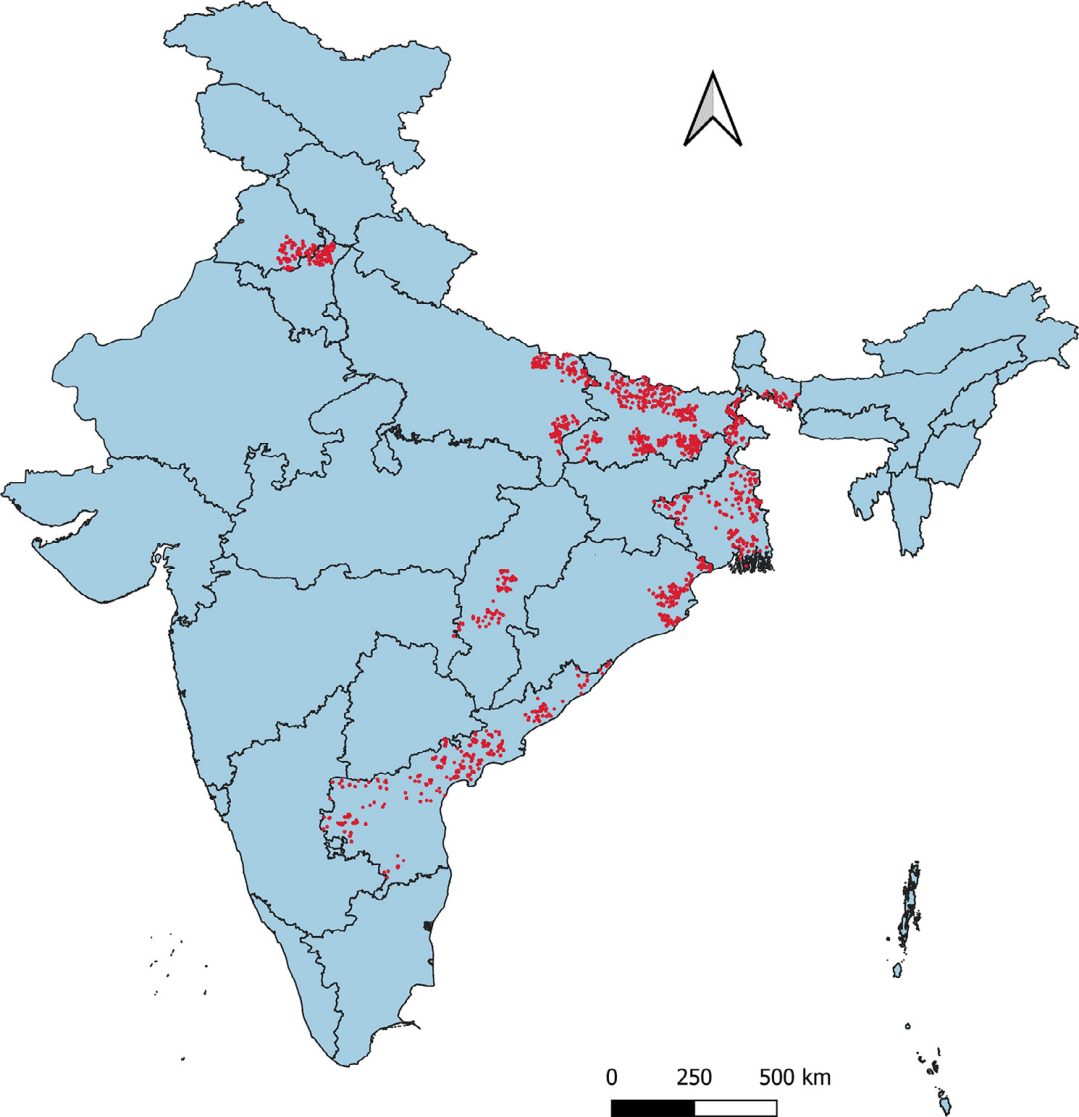
Indian map with state boundaries showing locations of rice survey through red dots.

Map 1 shows geo-locations of the survey on Indian map and a broader distribution within state boundaries. This map was developed using geo-coordinates of each surveyed rice plots captured at the end survey with QGIS Desktop App (version 3.22.7).

Table 1 describes how the samples were distributed across states, farmers’ typology, and characteristics of surveyed rice plots. It shows that maximum number of samples were from Bihar followed by West Bengal; Punjab and Haryana states had limited samples. Percentage of female farmers surveyed was 3.7% of the total sample. Educational status of sampled farmers depicts that one in four had never attended a school. Majority of the farmers were either from Other Backward Caste (OBC) or from General category. Most of the surveyed plots were owned by interviewed farmers. The soil type of these plots was mostly medium textured and water retention capacity of these plots was also in the middle range. This table was drawn using Rstudio (version 1.4.1106), an open-source data analytical tool.

**Table 1:** Distribution of samples by states, gender, education and caste along with typologies of surveyed rice plots.

No	Variable	States/Values	Freqs (% of Valid)	Valid	Missing
1	State [character]	1. Andhra Pradesh 2. Bihar 3. Chhattisgarh 4. Haryana 5. Odisha 6. Punjab 7. Uttar Pradesh 8. West Bengal	1333 (16.0%) 2428 (29.1%) 505 (6.0%) 417 (5.0%) 784 (9.4%) 388 (4.6%) 964 (11.5%) 1536 (18.4%)	8355 (100%)	0 (0%)

2	Gender of farmer [character]	1. Female 2. Male	308 (3.7%) 8047 (96.3%)	8355 (100%)	0 (0%)

3	Education of farmer [character]	1. Bachelors 2. Masters 3. Matriculation 4. No Schooling 5. Primary 6. Senior Secondary	628 (7.5%) 130 (1.6%) 1667 (20.0%) 2143 (25.6%) 2953 (35.3%) 834 (10.0%)	8355 (100%)	0 (0%)

4	Caste of farmer [character]	1. General 2. OBC 3. Other 4. SC 5. ST	3211 (38.4%) 3516 (42.1%) 6 (0.1%) 1352 (16.2%) 270 (3.2%)	8355 (100%)	0 (0%)

5	Land ownership [character]	1. Contract 2. Leased 3. Owned	97 (1.2%) 1382 (16.5%) 6876 (82.3%)	8355 (100%)	0 (0%)

6	Soil texture of fields [character]	1. Heavy 2. Light 3. Medium	1140 (13.6%) 618 (7.4%) 6597 (79.0%)	8355 (100%)	0 (0%)

7	Drain class of fields [character]	1. Lowland 2. Medium Land 3. Upland 4. Very Lowland	1480 (17.7%) 6025 (72.1%) 660 (7.9%) 190 (2.3%)	8355 (100%)	0 (0%)

Table 2 provides information on rainy season calendar months and corresponding rainfall in millimeters (mm) for surveyed states [13]. It also highlights rainfall received during this rainy season as a percent of total annual rainfall (mm) of the year 2018.

**Table 2:** Rainfall (mm) received in the surveyed states during rainy season calendar months of 2018.

			Rainy season months and rainfall (mm)					

Sr. No.	States	Annual rainfall (mm)	JUN	JUL	AUG	SEP	Rainy season rainfall (mm)	Rainy season rainfall (% of annual)
1	Andhra Pradesh	663.8	90.5	117.1	141.3	97.7	446.6	67%
2	Bihar	860.6	100.3	291.5	266.3	112.8	770.9	90%
3	Chhattisgarh	1211.9	159.3	381.6	419.5	143.8	1104.2	91%
4	Haryana	478.4	71.1	156.1	78.6	130.3	436.1	91%
5	Odisha	1630.0	155.3	434.7	413.8	286.3	1290.1	79%
6	Punjab	629.0	95.5	166.2	105.3	202.1	569.1	90%
7	Uttar Pradesh	805.4	40.8	299.1	293.1	132.4	765.4	95%
8	West Bengal	1444.1	260.1	358.7	269.2	239.5	1127.5	78%

**Fig. 1:**
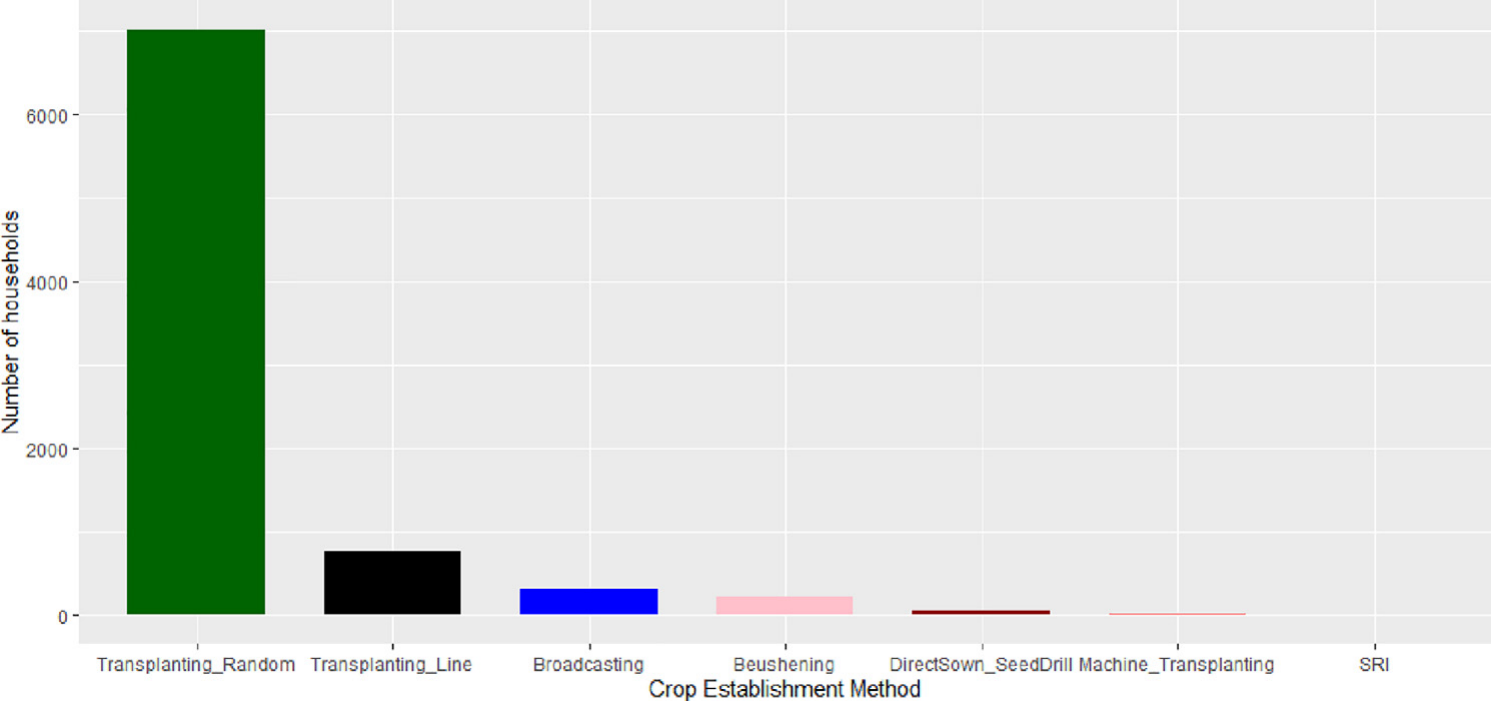
Different types of rice establishment methods applied by respondent farmers on their largest plot.

Fig. 1 depicts methods practiced by farmers to establish rice crop in their largest plot. Farmers were found to practice seven different ways to trans(plant) rice. These were transplanting seedling randomly, transplanting seedling in line, broadcasting seed on wet field, broadcasting higher rate of seed followed by uprooting poor seedlings almost a month later (*beushening*), sowing seed with seed drill machine, transplanting specially grown seedling with machine, and through system of rice intensification method. Out of seven different methods, the most frequent method was transplanting seedling randomly. It was found with 84% of the sample followed by transplanting seedling in line by 9% farmers.

**Fig. 2:**
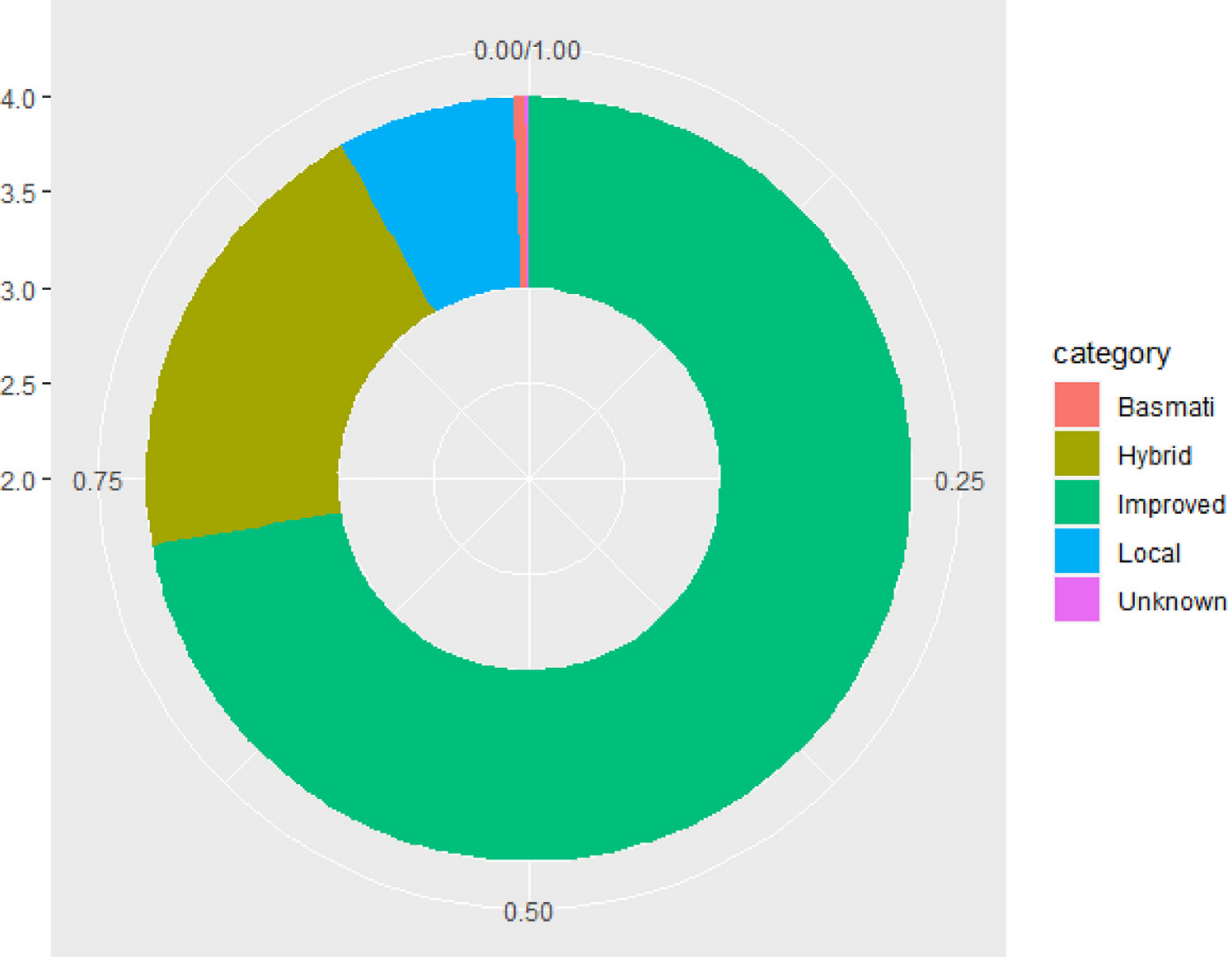
Categories of rice varieties used by respondent farmers on their largest plot.

Fig. 2 showcases various categories of rice seeds used by farmers. 72% of surveyed farmers were found to be using improved open pollinated varieties followed by 20% using rice hybrids. Occurrence of Basmati (scented) group of varieties was limited to Haryana and Punjab states.

**Fig. 3:**
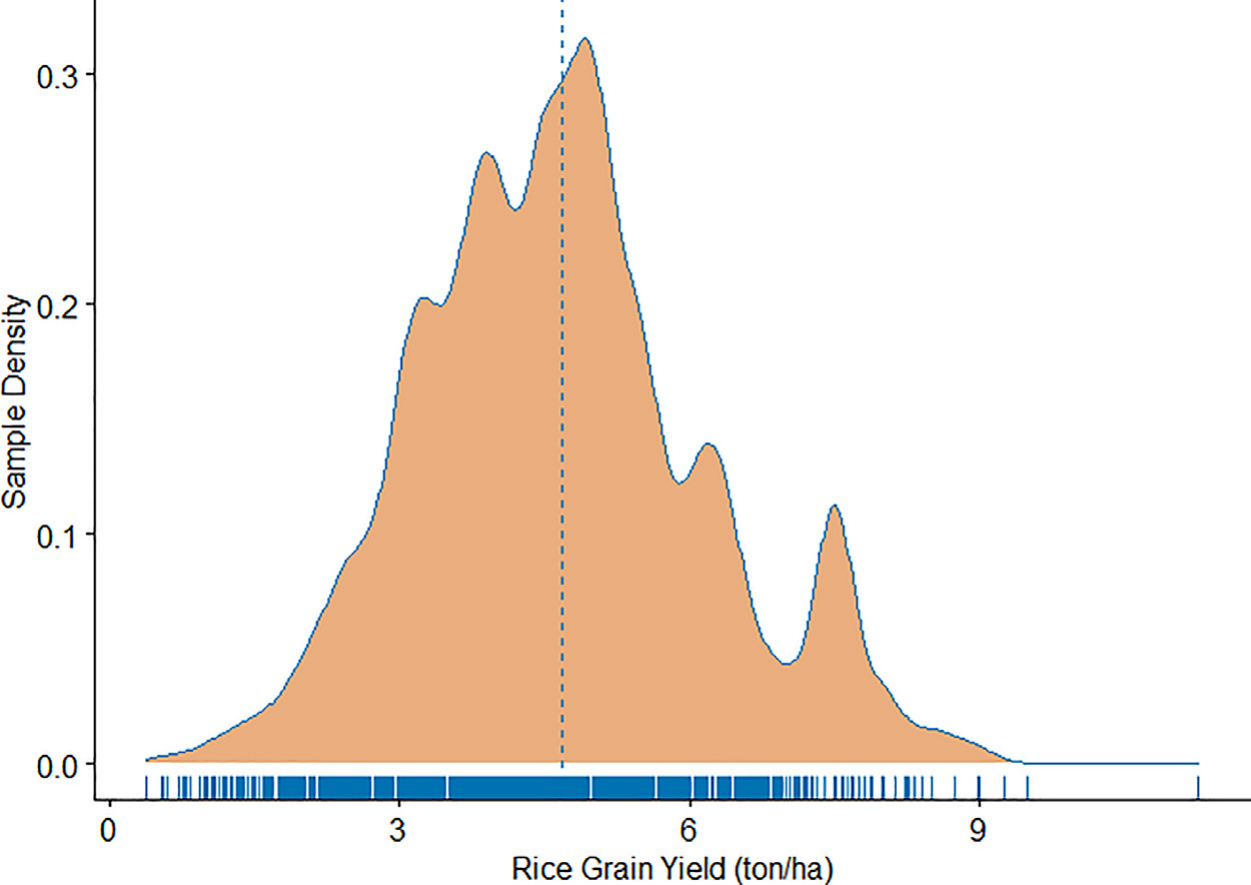
Distribution of grain yield on largest rice plot as reported by respondents (vertical blue line indicates mean yield of the sample).

Fig. 3 shows distribution of overall rice grain yields as reported by surveyed farmers through a density plot, smoothed version of the histogram [7]. It shows that the mean rice yield of sampled farmers was 4.7 tons per hectare as indicated by dotted vertical blue line. This figure was drawn using ‘ggpubr’ package of Rstudio.

**Fig. 4:**
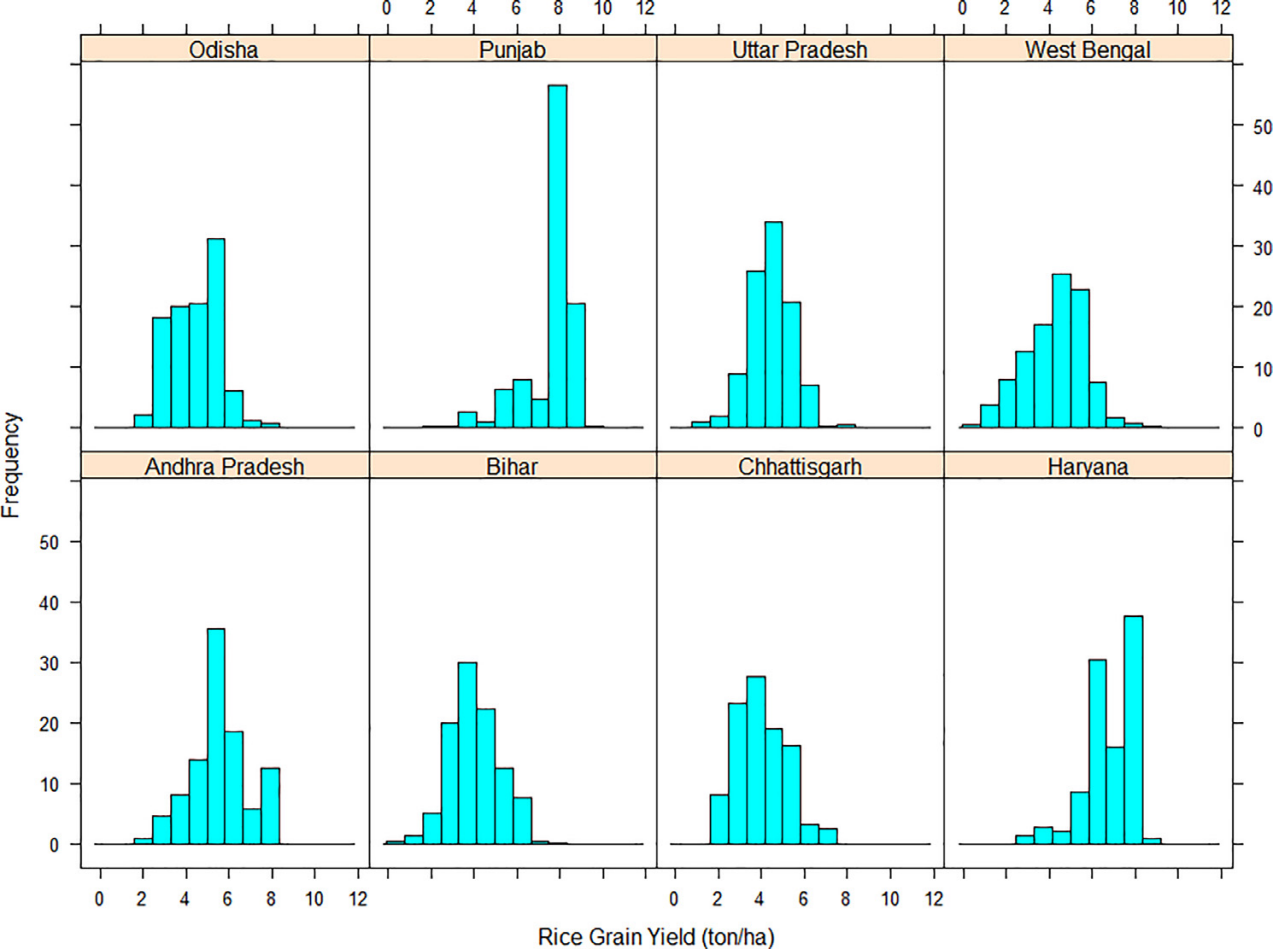
Rice grain yield histogram showing its distribution segregated by states.

Fig. 4 presents distribution of rice grain yields by states through merged histograms drawn with ‘ggplot2’ package of Rstudio [5]. It shows concentration of samples on the right side (higher yield levels) of the plot for states like Punjab and Haryana. For states such as Chhattisgarh, Odisha and Bihar, yield samples were more towards left side of the respective plots denoting lower rice yields of most farmers.

## 2 Experimental Design, Materials and Methods

District was taken as one survey unit and so sampling frames were constructed for all 49 districts separately. Single stage cluster sampling (a type of probability sampling) approach was followed to select farmers in districts. In the first stage, 30 villages were selected randomly in each district using probability-proportionate-to-size (PPS) where size refers to number of households in the village. PPS method is well suited when population of sampling units (villages in this case) vary in size [12]. It reduced standard error and bias by increasing the likelihood that a sampling unit from a larger population will be chosen over a sampling unit from a smaller population. After village selection was made, seven households were selected randomly in each village for conducting personal interviews. Accordingly, ideal sample size for each survey unit (district) was 210.

### 2.1 Village Selection

To construct sampling frame for village selection, census data of India 2011 [4] available in the public domain was used. All villages within a district were listed where corresponding number of households were also known. Extremely small (villages having <50 households) and extremely large (villages having >5000 households) were discarded along with villages categorized as urban habitat [10]. Final village list was accordingly generated to apply PPS. Steps followed to draw 30 villages in a district:

• In column next to number of households, generate cumulative number of households starting from number ‘1’. Last row in this column should match with total number of households (say N) in the sampling frame of villages.
• Add another column that calculate range of cumulative numbers generated before with respect to each village in the list. For example, if first village has 80 households, this column will show 1–80 and if second village has 50 households, this column will show 81–130. The last cell of this column should have something like ‘x’ – N.
• In any blank cell of this Excel sheet, type the formula =RANDBETWEEN (1, N). Run this formula to generate a random number. If we get 112, we draw second village as 112 falls between the range of 81–130 designated for second village.
• Repeat generation of random numbers by pressing F9 key to complete 30 unique selection.

### 2.2 Household Selection

Seven households were selected in 30 villages each through simple random sampling [11]. To make this selection, a list of villagers was generated through voter list available on the election commission websites of the respective states [8]. House number attached to each voter was treated as one household [10]. For example, if house number 71 was imprinted with six different names in the voter list, they were treated as one household.

**Map 2:**
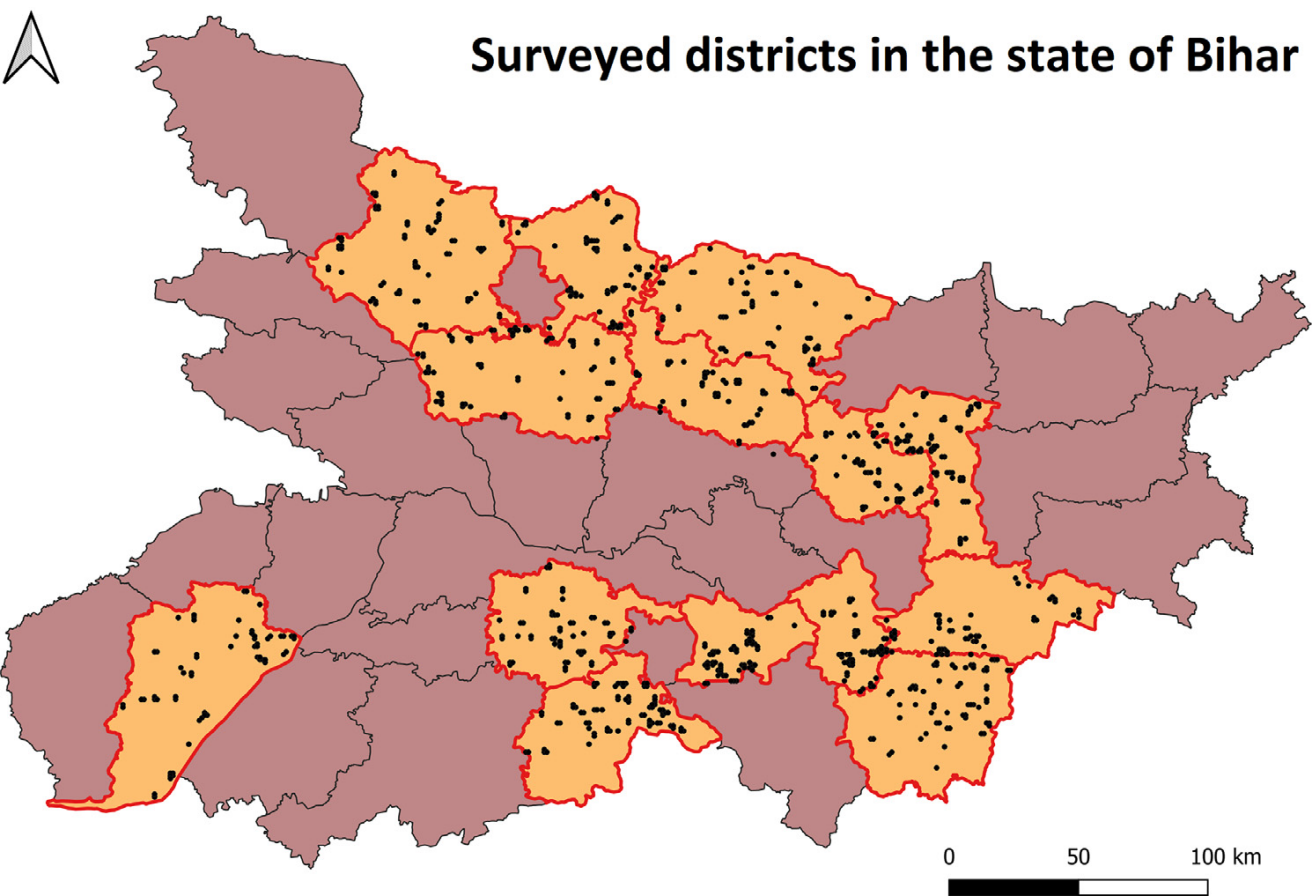
Map of Bihar state (one of the surveyed states) highlighting districts covered in the survey with red boundaries and sample distribution within districts by black dots.

Map 2 illustrates sample distribution (locations of farmers’ largest rice plots surveyed) in 14 Districts in Bihar. This map clearly depicts that sampling methodology applied in this survey generated uniformly distributed samples within a district.

### 2.3 ODK Collect Application

Trainings were conducted for enumerators separately in each state to set-up their Android devices with ODK server, discuss survey questions and explain the sampling frame (list of villages and house numbers/member names). Mock interviews were organized on the second day of the training. ODK Collect app [9] was downloaded on enumerator's device and linked to ODK server hosted in New Delhi by Indian Agriculture Statistics Research Institute (IASRI). Credentials were given to enumerators so that they can download digital survey Form [6] namely ‘Landscape Diagnostic Survey’ from server and deploy. Completed survey Forms were sent to the server by enumerators.

## Ethics Statements

The survey was conducted under Cereal Systems Initiative for South Asia (CSISA) project of International Maize and Wheat Improvement Center (CIMMYT). The project took formal approval of CIMMYT's Internal Research Ethics Committee (IREC) to collect and use farmers’ data. Before each farmer's interview, we clarified respondent the purpose and use of the data. All interviewees gave their prior informed consent to participate in the survey and were informed that they could withdraw at any point in case. Dataset was adequately anonymized so that neither individual participant nor their surveyed farm plot can be identified.

## CRediT authorship contribution statement

**Anurag Ajay:** Writing – original draft, Software. **Peter Craufurd:** Writing – review & editing. **Virender Kumar:** Writing – review & editing. **Arindam Samaddar:** Methodology. **RK Malik:** Project administration, Data curation. **Sachin Sharma:** Data curation. **Harshit Ranjan:** Methodology. **AK Singh:** Supervision. **Gokul Paudel:** Visualization. **Ajay Kumar Pundir:** Investigation. **Shishpal Poonia:** Investigation. **Anurag Kumar:** Investigation. **Pankaj Kumar:** Investigation. **Deepak Kumar Singh:** Investigation. **Madhulika Singh:** Investigation. **Wasim Iftikar:** Investigation. **Moben Ignatius:** Investigation. **Narayan Banik:** Investigation. **Bidhan Mohapatra:** Investigation. **Pardeep Sagwal:** Investigation. **Ashok Kumar Yadav:** Supervision. **Sugandha Munshi:** Supervision. **Peramaiyan Panneerselvam:** Supervision. **Andrew McDonald:** Conceptualization.

## Declaration of Competing Interest

The authors declare that they have no known competing financial interests or personal relationships that could have appeared to influence the work reported in this paper.

## Data Availability

Large-scale data of crop production practices applied by farmers on their largest rice plot during 2018 in eight Indian states (Original data) (Dataverse).
